# Valproic Acid Prevents Renal Dysfunction and Inflammation in the Ischemia-Reperfusion Injury Model

**DOI:** 10.1155/2016/5985903

**Published:** 2016-04-18

**Authors:** Elerson C. Costalonga, Filipe M. O. Silva, Irene L. Noronha

**Affiliations:** ^1^Laboratory of Cellular, Genetic, and Molecular Nephrology, Renal Division, University of São Paulo, 01246-903 São Paulo, SP, Brazil; ^2^Center for Cellular and Molecular Studies and Therapy (NETCEM), University of São Paulo, 01246-903 São Paulo, SP, Brazil

## Abstract

Ischemia-reperfusion injury (IRI) is a major contributor to acute kidney injury (AKI). At present, there are no effective therapies to prevent AKI. The aim of this study was to analyse whether valproic acid (VPA), a histone deacetylase inhibitor with anti-inflammatory properties, prevents renal IRI. Male Wistar rats were divided into three groups: SHAM rats were subjected to a SHAM surgery, IRI rats underwent bilateral renal ischemia for 45 min, and IRI + VPA rats were treated with VPA at 300 mg/kg twice daily 2 days before bilateral IRI. Animals were euthanized at 48 hours after IRI. VPA attenuated renal dysfunction after ischemia, which was characterized by a decrease in BUN (mg/dL), serum creatinine (mg/dL), and FENa (%) in the IRI + VPA group (39 ± 11, 0.5 ± 0.05, and 0.5 ± 0.06, resp.) compared with the IRI group (145 ± 35, 2.7 ± 0.05, and 4.9 ± 1, resp.; *p* < 0.001). Additionally, significantly lower acute tubular necrosis grade and number of apoptotic cells were found in the IRI + VPA group compared to the IRI group (*p* < 0.001). Furthermore, VPA treatment reduced inflammatory cellular infiltration and expression of proinflammatory cytokines. These data suggest that VPA prevents the renal dysfunction and inflammation that is associated with renal IRI.

## 1. Introduction

The incidence of acute kidney injury (AKI) is increasing and it is associated with high mortality and healthcare costs [1, 2]. Despite an increase in the knowledge of AKI pathogenesis and epidemiology, AKI remains without any effective treatment. Renal ischemia-reperfusion injury (IRI) is a major contributor to AKI in different clinical conditions. In some of these conditions, such as transplantation, aortic surgeries, and other major surgeries, there is a favourable period of time to implement preventive measures. It seems reasonable to assume that preventing AKI can avoid a significant number of deaths.

IRI induces tubular epithelial cell dysfunction and death through apoptosis and necrosis. Local hemodynamic changes, endothelial injury, and inflammation represent hallmarks of ischemic injury of the kidney [3]. In this setting, damaged and necrotic tubular epithelial cells (TECs) play a central role by actively inducing the production of cytokines and other inflammatory mediators (TNF-*α*, MCP-1, and IL-6), leading to the accumulation of inflammatory cells, which in turn worsen cell necrosis in an autoamplification loop process [4]. In AKI, the blockade of the initial inflammatory renal response is associated with decreased cytokine production and inflammatory cell infiltration that hampers kidney injury following IRI [5–8]. Thus, interventions that ameliorate tubular necrosis, apoptosis, and inflammation are promising for the prevention of AKI secondary to IRI.

Acetylation of histone and nonhistone proteins is well known to regulate gene transcription and cell signalling pathways involved in inflammation and apoptosis. Two enzymes, histone acetyl transferase (HAT) and histone deacetylase (HDAC), mediate this process [9]. HDAC inhibitors (HDACi) are promising therapeutic options for inflammatory diseases [10, 11]. The anticonvulsivant valproic acid (VPA) has been recognized as an HDAC inhibitor [12]. VPA inhibits nuclear factor *κ*-B (NF*κ*-B), TNF-*α*, and interleukin-6 (IL-6) production in human monocytic leukaemia cells stimulated with lipopolysaccharide [13]. VPA also reduced the inflammatory response and oxidative stress in septic mice in a model of caecum ligation and puncture, thereby protecting against renal injury [14]. In rats undergoing AKI due to haemorrhagic shock, the administration of VPA improved animal survival and inhibited the apoptosis of kidney cells [15]. VPA is an attractive HDACi because it has been clinically used for decades to treat epilepsy and mood disorders; it is cheap and available on the market.

Given that VPA modulates important pathways involved in AKI, we hypothesized that VPA might prevent the kidney dysfunction and inflammation that is induced in ischemia-reperfusion injury. Thus, the aim of this study was to evaluate the efficacy of VPA administration as a preventive intervention in an experimental renal IRI model. Specifically, we evaluated the ability of VPA to modulate inflammatory mediators and apoptosis in the injured kidney.

## 2. Materials and Methods

### 2.1. Animals

The experiments described were conducted using male Wistar rats weighing from 250 g to 300 g that were obtained from an established colony at the University of São Paulo, Brazil. The animals were housed in rodent cages in a 22°C room on a 12-hour light-dark cycle with free access to standard rat chow and water. All of the procedures received the approval of the Ethical Research Board of the University of São Paulo, Brazil (approval number: 381/13).

### 2.2. IRI Model and Study Design

All of the surgery procedures were performed under anaesthesia via the intraperitoneal administration of xylazine (36 mg/kg) and ketamine (5.7 mg/kg). During all of the procedures, animals were kept on a heat controlled thermal pad to maintain body temperature. Briefly, after a midline abdominal incision and careful dissection of the renal hilum, occlusion of the renal arteries of both kidneys with atraumatic vascular clamps for 45 minutes was performed to induce the experimental IRI. Clamps were removed and a remarkable change in the renal colour (pale to bright red) was observed ensuring good reperfusion. Animals were placed in heated cages until complete recovery from anaesthesia. Animals (*n* = 26) were divided into 3 groups, where SHAM (*n* = 6) rats were subjected to a SHAM surgery, IRI (*n* = 10) rats underwent IRI surgery and received vehicle administration, and IRI + VPA (*n* = 10) rats were treated with VPA (Abbot, Chicago, USA) at 300 mg/kg by gavage, twice daily, 2 days before the induction of IRI.

Animals were euthanized (pentobarbital sodium 100 mg/kg) at 48 hours after IRI, and then blood and kidney tissue samples were collected. The kidneys were perfused* in situ* and excised, and a midcoronal section was fixed in 10% phosphate buffered formalin. The other kidney sample was snap frozen and stored at −80°C for molecular analyses. One day before euthanasia, rats were placed in metabolic cages to collect 24-hour urine samples.

### 2.3. Biochemical Analysis

Serum and urinary creatinine (Cr), blood urea nitrogen (BUN), sodium, and potassium levels were measured using a Cobas C111 analyzer (Roche, Indianapolis, IN, United States). Fractional excretion of sodium (FeNa) and fractional excretion of potassium (FeK) were calculated. Urinary protein excretion was measured using a colorimetric assay (Labtest, Lagoa Santa, Brazil).

### 2.4. Acute Tubular Necrosis Grade

Kidneys sections (3 *μ*m) were stained with Periodic-Acid Schiff (PAS). Markers of tubular damage (tubular dilation, necrosis, and cast formation) were graded by calculation of the percentage of tubules in the cortex and corticomedullary junction presenting these features [16]. The scores were as follows: 0, none; 1, 1%–10%; 2, 11%–25%; 3, 26%–45%; 4, 46%–75%; and 5, >75%. Twenty microscopic fields (×200) of the kidney cortex and corticomedullary junction from each animal were analysed blind to the groups.

### 2.5. TUNEL Apoptosis Assay

The apoptosis of tubular cells was evaluated using nonradioactive TdT-mediated fluorescein-dUTP nick end labelling technique (TUNEL; ApopTag Peroxidase* in situ* Apoptosis Detection Kit Millipore Corporation, Billerica, MA, USA) on paraffin-embedded tissue. Briefly, paraffin sections were deparaffinised, digested with proteinase K (20 g/mL), and incubated with hydrogen peroxidase to block endogenous peroxidase activity. After washing, slides were incubated with the TdT enzyme, then incubated with antidigoxigenin peroxidase, and developed using a substrate containing diaminobenzidine. Negative controls included the omission of TdT. Positive apoptotic cells presented a strong brown nuclear reactivity [17]. The number of apoptotic cells per high power field (×200) from 20 sections of corticomedullary junction was obtained from each animal, and the results were expressed as mean number of positive cells/mm^2^.

### 2.6. Immunohistochemistry

Immunohistochemistry for inflammatory cells was carried out as previously described [18]. Paraffin-embedded renal biopsy specimens were processed for immunohistochemistry, and heating in citrate buffer, pH 6.0, was performed for antigenic recovery. After rinsing with pH 7.6 Tris-buffered saline (TBS), endogenous peroxidase activity was blocked and slides were incubated at 4°C overnight with the following monoclonal antibodies diluted in the primary antibody solution: anti-rat ED1 (Serotec, Oxford, UK) and anti-rat CD43 (Serolab, Oxford, UK). Both antibodies were diluted at 1 : 200. After primary antibodies incubation, the slides were submitted to another reaction with rat-biotinylated anti-mouse IgG (Vector Labs, Burlingame, USA) or with biotinylated anti-rabbit IgG (Vector Labs, Burlingame, USA). The streptavidin-biotin-alkaline phosphatase complex reaction was performed (Dako Co., Denmark). Finally, sections were incubated with a freshly prepared substrate consisting of naphthol-AS-MX-Phosphate (Sigma Chemical Co., St. Louis, USA) and fast red dye (Sigma Chemical Co., St. Louis, USA). Cellular proliferation was analysed using immunohistochemistry for proliferating cell nuclear antigen (PCNA) as previously described [19]. Quantitative analysis of macrophages (ED1), T-cells (CD43), and cellular proliferative activity was performed in a blind fashion under magnification (×200) and was expressed as cells/mm^2^. For each section, 20 microscopic fields were examined, and the results were expressed as the mean number of positive cells/mm^2^.

### 2.7. Analyses of Inflammatory Mediators in Kidney Tissue

The mRNA expression of TNF-*α*, IL-6, and MCP-1 in kidney samples was analysed using quantitative PCR as previously described [18]. In brief, total RNA was extracted from kidney samples using the TRIZOL Reagent (Ambion, Austin, TX). After total RNA reverse-transcription into cDNA, quantitative PCR was carried out. Reactions were performed using the StepOne Plus Real-Time PCR system, and quantitative comparisons were obtained using the ΔΔCT method (Applied Biosystems, Singapore). The RT-PCR cycle profile was 10 min at 95°C, followed by 40 cycles of 15 s at 95°C for denaturation, 20 s at 60°C for combined annealing, and 10 s at 72°C for extension. The primer sequences for amplifying the target genes were (forward) 5′-TGGCCCAGACCCTCACACTCA-3′ and (reverse) 5′-GGCTCAGCCACTCCAGCTGC-3′ for TNF-*α*, (forward) 5′-CCGGAGAGGAGACTTCACAGAGGA-3′ and (reverse) 5′-AGCCTCCGACTTGTGAAGTGGTATA-3′ for IL-6, and (forward) 5′-GCCCCACTCACCTGCTGCT-3′ and (reverse) 5′-TCTTTGGGACACCTGCTGCTGG-3′ for Monocyte Chemoattractant Protein (MCP-1). The 18S rRNA was used as a housekeeping control (forward 5′-AGTCCCTGCCCTTTGTACACA-3′ and reverse 5′-GCCTCACTAAACCATCCAATCG-3′). The levels of TNF-*α* were measured in protein extracts of kidney tissue samples using a commercial MILLIPLEX® MAP Kit (Millipore Corporation, Billerica, MA, USA).

### 2.8. Serum Thiobarbituric Acid Reactive Substances

To evaluate systemic oxidative stress, serum levels of thiobarbituric acid reactive substances (TBARS) were measured using the thiobarbituric acid assay [20]. Briefly, a 0.2 mL serum sample was diluted in 0.8 mL of distilled water. Next, 1 mL of 17.5% trichloroacetic acid was added. Following the addition of 1 mL of 0.6% thiobarbituric acid, the sample was placed in a boiling water bath at 100°C for 15 min. After cooling, 1 mL of 70% trichloroacetic acid was added, and the mixture was incubated for 20 min. The sample was then centrifuged for 15 min at 2,000 rpm. The optical density of the supernatant was read at 534 nm using a spectrophotometer. The concentration of malondialdehyde (MDA) was calculated using a molar extinction coefficient of 1.56 × 10^5^ M^−1^ cm^−1^ and was expressed as nmol/L.

### 2.9. Statistical Analysis

Data are presented as mean ± standard error of mean, and statistical analyses were performed with the Prism statistical program (GraphPad, San Diego, CA, USA). A one-way ANOVA with* post hoc* (Tukey) correction was used to compare all of the groups. To study the correlation between NTA grade, macrophages, and PCNA positive cells, a Pearson correlation coefficient was calculated. A *p* value < 0.05 was considered statistically significant.

## 3. Results

### 3.1. Valproic Acid Prevented the Kidney Dysfunction Induced by IRI

In the IRI group, BUN and serum creatinine levels were significantly higher than the SHAM group. VPA therapy attenuated the renal dysfunction induced by IRI, as shown by the significantly lower BUN and creatinine levels in the IRI + VPA group (Table 1). As expected, FeNa and FeK were increased in the IRI compared with the SHAM group. Notably, tubular function was protected by VPA administration (Table 1).

### 3.2. Valproic Acid Protected Acute Tubular Necrosis and Apoptosis

**Figure 1:**
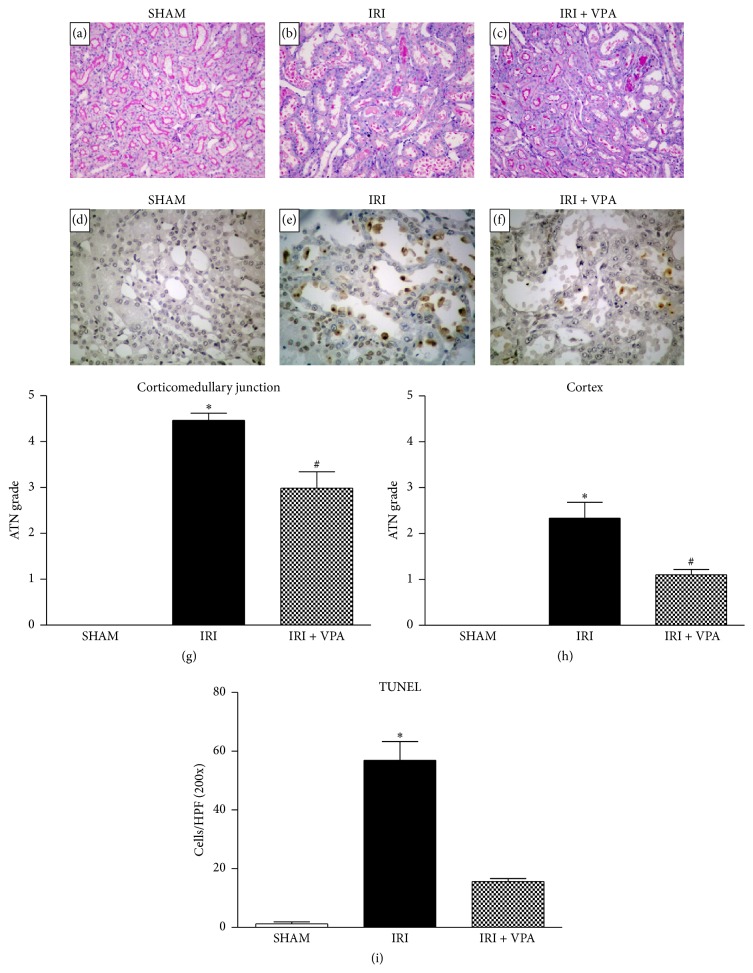
Effects of VPA in kidney acute tubular necrosis and apoptosis. (a)–(c) Representative photomicrographs of kidney sections stained with PAS (×200). (a) SHAM group. (b) IRI group showing an intense acute tubular necrosis (ATN). (c) In the IRI + VPA group, ATN was attenuated. (d)–(f) Representative photomicrographs of kidney sections (×400) stained with the TUNEL technique. (d) SHAM group. (e) IRI group showing a high number of apoptotic cells characterized by the presence of apoptotic bodies identified by brown staining. (f) VPA treatment attenuated apoptosis. (g, h) Graphs presenting the ATN grades in all of the groups. (i) Graph presenting the quantification of apoptotic cells in all of the groups. Results are expressed as mean ± standard error of mean. ^*∗*^ *p* < 0.001 versus SHAM and IRI + VPA. ^#^ *p* < 0.001 versus SHAM.

The IRI model induced necrosis and detachment of tubular epithelial cells, hyaline casts, and tubular dilatation, resulting in a significant grade of acute tubular necrosis (ATN, Figure 1). When compared with the IRI group, the ATN associated with IRI injury was significantly attenuated in animals treated with VPA, in both cortico (4.4 ± 0.1 versus 2.9 ± 0.4; *p* < 0.001) and corticomedullary junctions (2.3 ± 0.3 versus 1.1 ± 0.1; *p* < 0.001, Figure 1). Apoptosis, an important process of tubular cell death in renal IRI, was evaluated using a TUNEL assay. The number of apoptotic cells was significantly increased in the IRI group, although treatment with VPA prevented the apoptosis of tubular cells (57 ± 6 versus 15 ± 1 cells/high power field; *p* < 0.001; Figure 1).

### 3.3. Valproic Acid Prevented Leukocyte Infiltration and Cellular Proliferation

**Figure 2:**
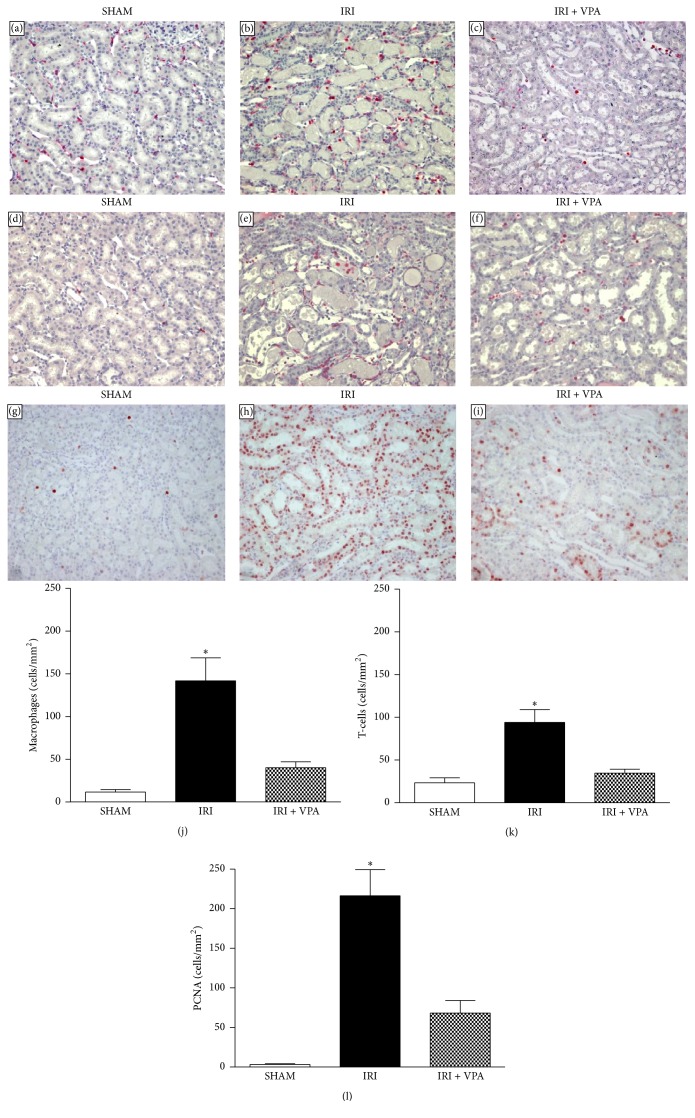
Immunohistochemistry for macrophages, T-cells, and proliferating cell nuclear antigen (PCNA). Representative photomicrographs (×100) of immunohistochemistry for macrophages (a)–(c), T-cells (d)–(f), and cellular proliferation (g)–(i). (a, d, g) SHAM groups. In the IRI group, an increased number of macrophages (b) and T-cells (e) were observed infiltrating the renal interstitium. Cellular proliferation activity was also markedly enhanced in the IRI group (h). VPA treatment significantly attenuated the number of mononuclear inflammatory cells and proliferative activity in the IRI model (c, f, i). Graph presenting the quantification of macrophages (j), T-cells (k), and PCNA (l).  ^*∗*^ *p* < 0.001 versus SHAM and IRI + VPA.

Inflammatory infiltration in the renal interstitial compartment constitutes one important feature of IRI. In the SHAM group, few inflammatory cells were detected. However, there was a striking number of macrophages (ED1^+^ cells) and T-cells (CD43^+^ cells) infiltrating the interstitium of the kidney in the IRI group (resp., ED1^+^ cells 13 ± 1 versus 141 ± 26 cells/mm^2^, *p* < 0.001; T-cells 23 ± 5 versus 94 ± 15 cells/mm^2^, *p* < 0.001). Treatment with VPA significantly reduced cellular inflammatory infiltration (ED1^+^ cells 40 ± 7 cells/mm^2^; T-cells 35 ± 4 cells/mm^2^, *p* < 0.001) (Figure 2). A positive correlation was found between the number of interstitial macrophages and the ATN grade (*r* ^2^ = 0.65; *p* = 0.02). Furthermore, compared with the SHAM group, the IRI group showed a higher cellular proliferation, as measured by PCNA immunohistochemistry (3 ± 1 versus 216 ± 33 cells/mm^2^, resp.; *p* < 0.001). Moreover, IRI + VPA animals showed a lower number of PCNA positive cells (68 ± 15 cells/mm^2^, Figure 2). In addition, we found a positive significant correlation between the ATN grade and the number of PCNA positive cells (*r* ^2^ = 0.67; *p* = 0.003).

### 3.4. Valproic Acid Diminished the Upregulated Expression of Inflammatory Cytokines in the Kidney

**Figure 3:**
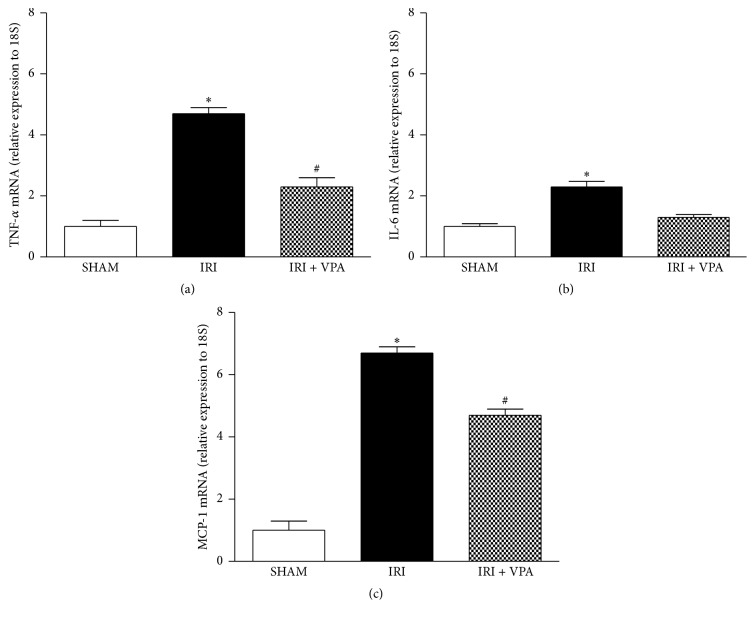
Renal expression of TNF-*α*, IL-6, and MCP-1 by qPCR in the IRI model. VPA prevented the increased expression of TNF-*α*, IL-6, and MCP-1 that is induced by IRI. ^*∗*^ *p* < 0.001 versus SHAM and IRI + VPA. ^#^ *p* < 0.001 versus SHAM.

To further explore whether VPA could reduce inflammation, real-time quantitative PCR (qPCR) for inflammatory cytokines, such as TNF-*α*, IL-6, and MCP-1, was performed (Figure 3). In the IRI group, mRNA levels of TNF-*α*, IL-6, and MCP-1 were significantly upregulated compared with the SHAM group. VPA treatment significantly reduced the gene expression of these cytokines in the renal tissue compared with the IRI group (*p* < 0.01, Figure 3). Moreover, in the IRI + VPA group, the increase of the TNF-*α* protein level induced by IRI was suppressed compared to the IRI group (SHAM group = 6.6 ± 0.4 versus IRI group = 10.0 ± 1.0 versus IRI + VPA group = 6.2 ± 0.7 pg/protein mg; *p* < 0.01).

### 3.5. Antioxidant Effect of Valproic Acid

**Figure 4:**
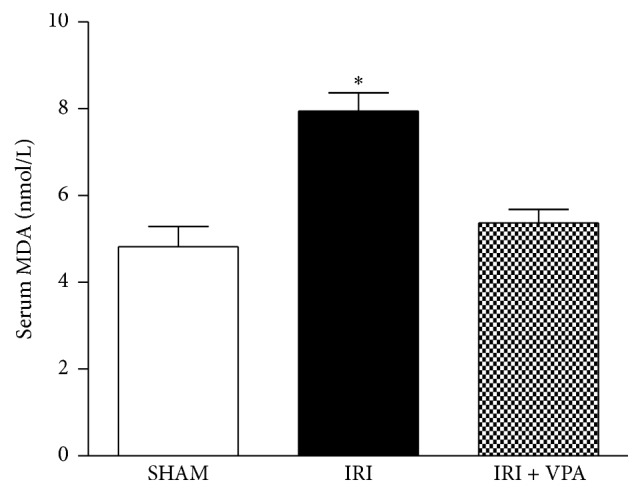
Antioxidant effect of VPA on the IRI model. Serum malondialdehyde (MDA), a marker of lipid peroxidation, was measured to evaluate the systemic oxidative stress in the IRI model. The IRI group showed a significant increase in the serum concentration of MDA. In contrast, the VPA treatment demonstrated an antioxidant effect. ^*∗*^ *p* < 0.001 versus SHAM and IRI + VPA.

Serum malondialdehyde (MDA), a marker of lipid peroxidation, was measured to evaluate systemic oxidative stress due to IRI. The IRI group showed a significant increase in the serum concentration of MDA. In contrast, VPA treatment resulted in an antioxidant effect (resp., 7.9 ± 0.4 versus 5.3 ± 0.3 nmol/L; *p* < 0.01). Notably, in the IRI + VPA group, the MDA concentration was maintained at a level similar to the control (Figure 4).

## 4. Discussion

The results of the present study demonstrated that administration of VPA was effective for preventing kidney dysfunction in the rat IRI experimental model. The rise in serum creatinine and BUN following IRI was reduced by VPA treatment. The tubular function was also protected, as confirmed by the lower FENa and FEK in VPA-treated animals compared to the IRI group. A marked decrease in acute tubular injury, apoptosis, and inflammation was also observed in IRI rats treated with VPA. The attenuation of all of these pathways may have contributed to the preservation of kidney function in the IRI + VPA group. Reduced levels of BUN and serum creatinine observed in VPA-treated animals in the present study are consistent with previous reports that investigated the effect of VPA in AKI, secondary to experimental haemorrhagic shock [15] and IRI [21]. Notably, no side effects such as cardiac arrest and sudden death, as described by Speir et al. [21], were observed in the present study in animals receiving VPA doses of 300 mg/kg twice daily. The absence of adverse effects was also reported in other experimental models using VPA at a similar dosage [15, 22–24].

Reduced acute tubular necrosis at 48 h after ischemia reperfusion confirmed the protective effect of VPA treatment. The actions of VPA in protection against IRI injury may be due to its ability to suppress inflammatory mediators [15, 21] and the oxidative stress [22, 25] that occurs following IRI. In addition, apoptosis of TECs is an important mechanism of cell death in renal IRI. VPA treatment in animals subjected to IRI inhibited the apoptosis of kidney cells, confirming previous findings [15, 21]. This effect is possibly related to the upregulation of the antiapoptotic BCL-2 expression [21]. These findings provide evidence of VPA preventing acute tubular necrosis and apoptosis in renal IRI.

In the present study, macrophages were the main interstitial inflammatory cells found 48 h after IRI. VPA treatment promoted a marked reduction of macrophages in the tubulointerstitial compartment. Likewise, VPA also reduced the macrophage infiltration in an adriamycin nephropathy model [26]. These results can be attributed to the inhibitory effect of VPA in the expression of inflammatory cytokines involved in leukocyte trafficking, such as TNF-*α* and MCP-1 [26, 27]. Another interesting finding was the positive correlation between the ATN grade and the number of macrophages, highlighting the interplay between inflammation and TEC necrosis. Necrotic TECs are considered to be important sources of inflammation that activate reminiscent tubular cells to produce cytokines that promote inflammatory cell infiltration [3]. Subsequently, the attenuation of macrophage infiltration may be the result of a lower tubular necrosis grade in the VPA-treated group.

The number of T-cells detected in the kidney at 48 h after IRI was also lower in animals treated with VPA. In the early period after IRI, CD4^+^ T-cells are the primary pathogenic T-cells. In nu/nu mice (CD4 and CD8 T-cell-deficient mice), renal IRI was significantly reduced compared to wild-type controls and reconstitution with CD4^+^ T-cells alone restored kidney injury [28]. Thus, the reduction of lymphocytes in the kidney of VPA-treated animals contributed to the protection of kidney function and structure.

The analysis of cellular proliferation by PCNA immunostaining showed that VPA-treated animals presented lower renal tubular cellular proliferation than the IRI + VPA group. There was also a significant positive correlation between ATN and cellular proliferation. After IRI there is an intense increase in TEC turnover to replace necrotic and apoptotic cells [3]. The results observed in the present study suggest that the reduction of cellular proliferation is secondary to a lower grade of ATN presented by rats receiving VPA. However, VPA may directly inhibit cellular proliferation. MS-275, a class I HDAC inhibitor similar to VPA, reduced the expression of PCNA in AKI that is induced by rhabdomyolysis [29]. Although VPA protected kidney function in the present study, the inhibition of cellular proliferation may have a detrimental effect on tubular regeneration.

There is increasing evidence of the anti-inflammatory effects of VPA [30]. In our experiments, we observed significantly lower expression of proinflammatory genes TNF-*α*, IL-6, and MCP-1 in the kidneys of VPA-treated animals compared to the IRI group, which confirmed previous reports [31, 32]. This effect may be due to the inhibition of NF-*κ*B signalling, a key transcription factor involved in inflammatory gene activation [31, 32]. Moreover, in early kidney IRI, macrophages synthesize TNF-*α*, which in turn stimulates inflammation and apoptosis [8], thereby promoting extension of the injury. The exposure to VPA significantly repressed the production of TNF-*α* in the mouse macrophage cell line RAW264.7 that was stimulated with LPS [33]. Thus, the inhibition of TNF-*α* production by VPA may account for its beneficial effect in kidney IRI.

In the present study, the increased MCP-1 mRNA expression induced by IRI in the kidney was attenuated by VPA pretreatment. In a previous report, the magnitude of renal IRI was diminished in mice lacking the receptor for MCP-1 [34]. Through unknown mechanisms, VPA was shown to reduce the expression of MCP-1 in different experimental models [26, 27]. Thus, the inhibition of MCP-1 expression by VPA may have hampered the macrophage infiltration preventing inflammation and extension of kidney damage.

VPA reduced lipid peroxidation, as evidenced by the lower levels of MDA found in the IRI + VPA group in comparison to the IRI group. Oxidative stress has an important role in the pathogenesis of ATN [3]. In previous studies, VPA administration prevented kidney dysfunction, reduced superoxide anion generation, and increased the activity of antioxidant enzymes after ischemic and septic kidney injuries [14, 25]. Hence, in this study, VPA may have prevented kidney dysfunction through antioxidant mechanisms.

It should be pointed out that the present study has some limitations. So far, the exact mechanism of VPA effects could not be entirely understood. As reported in previous studies, VPA might offer kidney protection by increasing histone acetylation and enhancing the expression of genes involved in apoptosis inhibition [21]. Considering that acetylation modulates the activity of nonhistone proteins, it can be hypothesized that VPA could modulate molecular activity of some transcription factors, such as NF-*κ*B. It has been shown that acetylation of p65 NF-*κ*B subunit reduces the binding of NF-*κ*B to promoter regions and facilitates its I*κ*B*α*-dependent nuclear export [35]. Furthermore, we studied the effect of VPA in the first 48 hours following kidney IRI. Whether the beneficial effect of VPA on kidney function will be maintained in the long term remains an open question and deserves further investigation. The present study confirmed previous findings about the beneficial effects of HDACi and VPA in the treatment of IRI.

## 5. Conclusion

**Figure 5:**
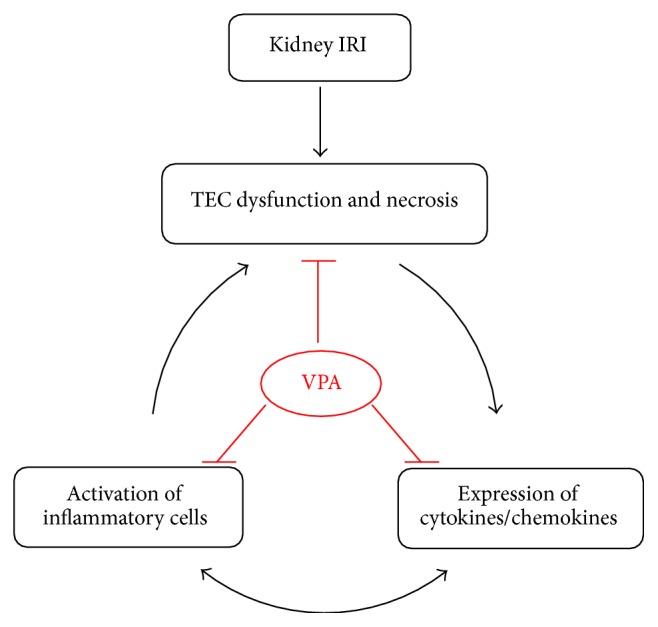
Possible effects of VPA on the kidney inflammation that is induced by ischemia-reperfusion injury. Acute kidney injury (AKI) following ischemia-reperfusion injury (IRI) leads to tubular epithelial cell (TEC) dysfunction, necrosis, and endothelial activation, which in turn induces the production of proinflammatory cytokines and infiltration of inflammatory cells into the kidney, in addition to an increase in the expression of cytokines and chemokines. This autoamplification loop of inflammation and tubular damage leads to an extension of kidney damage, despite the interruption of ischemia. In the IRI model, valproic acid (VPA) attenuated kidney dysfunction and acute tubular necrosis. These findings were associated with a marked decrease of macrophage and T-cell infiltration and proliferative activity and an inhibition of proinflammatory cytokines and chemokines (TNF-*α* and MCP-1).

In summary, AKI following IRI is associated with the production of proinflammatory cytokines and the infiltration of inflammatory cells into the kidney. This process leads to an extension of kidney damage despite a cessation of the ischemic stimuli. We have demonstrated that VPA prevented kidney dysfunction and structural injury after kidney IRI. VPA affected kidney inflammatory cell infiltration and the expression of important proinflammatory cytokines (TNF-*α* and MCP-1), demonstrating the anti-inflammatory effect of VPA on kidney IRI (Figure 5). VPA may represent a new treatment option for the prevention of kidney ischemia-reperfusion injury, but future studies are needed to investigate its efficacy and safety.

## Figures and Tables

**Table 1:** Effects of VPA on renal function and urinary biochemistry.

	SHAM (*n* = 6)	IRI (*n* = 10)	IRI + VPA (*n* = 10)
BUN (mg/dL)	13 ± 1	145 ± 35^*∗*^	39 ± 11
Creatinine (mg/dL)	0.2 ± 0.0	2.7 ± 0.4^*∗*^	0.5 ± 0.1
FENa (%)	0.2 ± 0.1	4.9 ± 1^*∗*^	0.5 ± 0.1
FEK (%)	22 ± 3	129 ± 17^*∗*^	17 ± 1.4
Urinary protein/creatinine	1.85 ± 0.2	3.9 ± 0.8	2.18 ± 0.2

BUN: blood urea nitrogen; FENa: fractional excretion of sodium; FEK: fraction excretion of potassium; IRI: ischemia-reperfusion injury; VPA: valproic acid.

Results are expressed as mean ± SEM. ^*∗*^ *p* < 0.001 versus SHAM and IRI + VPA.
